# Burn: A Predictable but Preventable Tragedy in Epileptic Patients

**DOI:** 10.29252/wjps.8.2.254

**Published:** 2019-05

**Authors:** Masoumeh Ghoddusi Johari, Ali Akbar Mohammadi, Vahid Dastgerdi

**Affiliations:** 1 Breast Disease Research Center, Shiraz University of Medical Sciences, Shiraz, Iran;; 2 Burn and Wound Healing Research Center, Plastic and Reconstructive Surgery Ward, Shiraz University of Medical Science, Shiraz, Iran

**Keywords:** Burn, Injury, Patient, Prevent, Epilepsy, Iran

## Abstract

**BACKGROUND:**

Epilepsy, the world’s most common neurological brain dysfunction, affects more than 50 million people worldwide. Burn injuries can be the leading cause of morbidity and mortality in the patients. This study assessed the predictable but preventable tragedy in epileptic burn patients.

**METHODS:**

From January 2001 to January 2011, data included patient’s demographic, burn cause, Total Body Surface Area (TBSA) of the burn injury, patient’s risk awareness, the type of treatment required as well as the treatment outcome were collected from burn admissions. Totally, 39 patients who sustained burn injuries due to epileptic seizures w were enrolled.

**RESULTS:**

Totally, 39 (1.7%) were epileptic with mean age of 30±11 years, 51.3% were female, 41.2% were single and 53.84% were rural residents and 12.8% had academic education. The majority of the thermal injuries occurred at home (82.1%). Flame was the most common cause of burn (66.7%). The mean Total Body Surface Area was 19.69±18.25. Finally, 38 patients were discharged with mortality rate of 2.6%. Thirty patients underwent split or full thickness graft as the most common surgery. Only 5 patients were aware of the burn injury risk during seizure attack.

**CONCLUSION:**

Despite reduction in burn injuries secondary to seizure, still such injuries lead to significant morbidity and mortality. Since these patients should adhere to specific medication, controlling it remains to be difficult. So preparation for preventive strategies is consisted of life style modification along with patients’ education that is further warranted.

## INTRODUCTION

Epilepsy, the world’s most common neurological brain dysfunction, affects more than 50 million people worldwide.^1^ This clinical manifestation is diagnosed by the development of two or more unprovoked seizure episodes,^2^ which might begin in first years of life, leading to serious disabilities.^3^ People with epilepsy are at risk of seizure-related injuries in comparison with the general population, especially in periictal period.^4^^,^^5^ The patient’s whose seizures have gone unmanaged are at higher risk.^6^ It is well known that during seizure attacks, patients might be afflicted by serious trauma like limb fractures, head and neck injuries and burns.^3^

Burn injuries are the leading cause of morbidity and mortality. Based on World Health Organization (WHO) report, East Mediterranean region is associated with the most disabling features, including psychological and functional incompetency, as a particular challenge in this population.^2^^,^^7^ The high incidence of burns amongst epileptic patients has long been perceived.^8^ Prolonged contact with heat might occur during the first minutes of unconsciousness as the preface of epileptic attack, and lead to severe and serious burn injuries. The most common risk factors reported are seizure frequency, noncompliance to the medications, and especially patient’s lack of attentiveness.^6^^-^^8^ To the best of our knowledge, this is the first study to be conducted in Iran on inadequate management of this disorder, leading to vulnerability of this group of patients. Hence, this calls for a preventive strategy to be designed along with targeting epileptic patients.

## MATERIALS AND METHODS

This study was conducted at the Shiraz Burn and Wound Healing Research Center affiliated to Shiraz University of Medical Sciences, Shiraz, Iran. Data were collected from the Burn admission forms from January 2001 to January 2011. Totally, 39 patients who sustained burn injuries due to epileptic seizures were enrolled. Data included patient’s demographic, burn cause, Total Body Surface Area (TBSA) of the burn injury, patient’s risk awareness, the type of treatment required (the type of surgery) as well as the treatment outcome, including mortality. 

## RESULTS

**Fig. 1:**
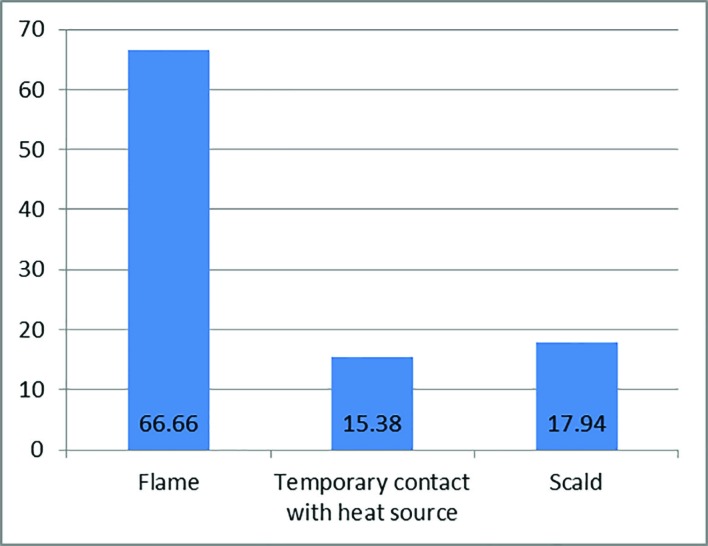
The mechanism of burn injury

**Fig. 2:**
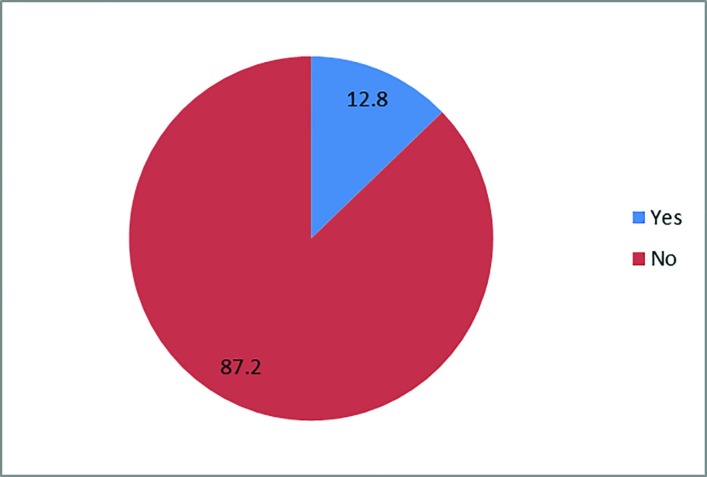
Risk factor awareness

Out of 2350 adult patients with burn injury, 39 (1.7%) were epileptic. A total of 51.3% of patients were female. The female/male ratio was 20:19. The mean age of patients was 30±11 years, 41.2% were single and married to single ratio was 23:16 and 53.84% of them were rural residents. Only 5 patients (12.8%) had academic education, while the illiterate to literate ratio was 34:5. The majority of the thermal injuries occurred at home (82.1%), followed by workplace (10.3%), and outdoor (7.7%). Based on the cause of injuries, flame was the most common one (66.7%) (Figure1). The mean Total Body Surface Area was 19.69±18.25. Finally, 38 patients were discharged, and a 41 year old man died due to sepsis, and the mortality rate was 2.6%. Out of 39 patients, 30 underwent different types of surgeries with the split or full thickness graft as the most common used technique. The injuries led to limb amputation of 3 patients. From 39 patients, only 5 were aware of burn injury risk during seizure attack (Figure 2). Out of 39 patients, 5 were readmitted to hospital due to burn injury post-seizure attack, indicating lack of awareness.

## DISCUSSION

Patients with seizure disorder are vulnerable group to burn injuries and might suffer during seizure attack.^4^^,^^5^ Several studies showed that epileptic patients were susceptible to higher rate of burns than the general populations.^2^^,^^6^^,^^9^ These patients should be identified to provide them with specific therapeutic measurers and train them on how to deal with unpredictable attack that might predispose them to burn injuries. Disorientation followed by seizure attack might lead to sever burn injuries, even mortality.^10^

Unintentional and unacceptable social behaviors might cause people to neglect these patients, leading to more vulnerability. Despite reduced rate of burn injuries amongst epileptic patients in the developed countries, it still remains high in the developing ones, indicating lack of proper management of epilepsy and burn injuries.^1^ The different prevalence of seizure related physical injuries were observed in several studies, as shown by Asadi-pooya *et al.* (47.3%, Iran).^11^ Burns as a consequence of epilepsy accounted 5-10 % in previous studies, and in this study, it was 1.66%.^4^^,^^10^

The main limitation of this study was that the outpatients’ who had referred to the burn center from 2001 to 2011 were not recorded. The mean age of patients in this study was 30±11 years, indicating the need for educational program to prevent these events. This study revealed flame as the most common cause of burn injury contrary to other studies such as Akhtar *et al.* who showed scald as the most frequent cause.^10^ From the total, 82.05% of all injuries occurred at home, indicating that home was a high risk place for these patients,while doing house chores, which was also shown in several other studies.^5^^,^^7^^,^^12^^,^^13^

Majority of patients in this study had low socioeconomic status and lived in places that lacked electricity or natural gas plumbing, and had to use alternative and risky sources for cooking proposes, including open fires. Several studies showed that open fire was the most common cause of injury and mortality, indicating the need for educational program to prevent such events.^6^^,^^8^ Also, extra safety precaution measures should be considered in patient’s work places; despite the fact that they are usually not involved in some restricted professions. For example, in industrial sites that people are more prone to electrical burns. Consequently, utilizing labor standards and security systems would reduce the frequency of such injuries.^14^

It should be noted that burn injuries following seizure attack can be reduced, even can be prevented with simple actions such as using fire guard, heat resistant refractory clothing, use of microwave rather than stove or cookers, do not use hair dryer, and install sensors in the showers in order to control water temperature.^6^^,^^10^ Also to prevent these injuries, epileptic patients should perform high risk activities under supervision. Several studies suggested that it would be better for epileptic patients to use rotatory valves instead of lever ones, to set water temperature, since this kind of tap might function, if a seizure attack occur as a result of sudden body movement.^15^

Despite some studies that presented gender as a predictor of burn injury in epileptic patients,^5^^,^^7^ in this study the frequency of burn injury in both genders were approximately the same (51.28% vs. 48.72%), showing the necessity to provide a comprehensive prevention program for both groups. Other studies showed that women were at higher risk, since they were involved in daily chores. The differences between our findings and other studies might be due to different sample sizes and various socioeconomic and cultural statuses of the studied population. In this study, only 12.82% of the patients were literate.

It is noteworthy that burn care management is a combination of conservative therapy in superficial burn injuries and surgical interventions for deep ones, leading to long hospitalization, imposing a significant burden of cost on the healthcare system as well as the individual.^3^^,^^4^^,^^10^ Another important factor is proper consumption of medication in this group of patients by rising patients’ compliance and regular visit to neurologists for adjusting the drug dosage. Neurology clinics are the best place to attract patients’ attention to these issues. In a study in Saudi Arabia, 4 epileptic patients had burn injuries during the month of Ramadan (ninth month of the Islamic calendar, observed by Muslims worldwide as the month of fasting) due to absence of drug intake because of fasting as a religious belief.^16^

Lack of awareness about the potential thermal hazards is an important factor that should be considered. Also, there is need to rise public health awareness toward epilepsy.^9^ The best way for epileptic patients is to prevent from being hurt in the first place by modifying their lifestyle as well as to comply with taking anti-epileptic drugs by their physician’s orders.^3^^,^^17^^,^^18^ Despite reduction in burn injuries secondary to seizure, still such injuries lead to significant morbidity and mortality. Since these patients should adhere to specific medication, controlling it remains to be difficult. So preparation for preventive strategies is consisted of life style modification along with patients’ education that is further warranted. 
